# Disease-associated synaptic scaffold protein CNK2 modulates PSD size and influences localisation of the regulatory kinase TNIK

**DOI:** 10.1038/s41598-020-62207-4

**Published:** 2020-03-31

**Authors:** Hanna L. Zieger, Stella-Amrei Kunde, Nils Rademacher, Bettina Schmerl, Sarah A. Shoichet

**Affiliations:** 1 Neuroscience Research Center, Charité-Universitätsmedizin Berlin, Charitéplatz 1, 10117 Berlin, Germany; 2 German Center for Neurodegenerative Diseases, 10117 Berlin, Germany; 3 Present Address: Interdisciplinary Institute for Neuroscience, CNRS, University of Bordeaux, UMR 5297, F-33000 Bordeaux, France

**Keywords:** Molecular neuroscience, Epilepsy, Neurodevelopmental disorders

## Abstract

Scaffold proteins are responsible for structural organisation within cells; they form complexes with other proteins to facilitate signalling pathways and catalytic reactions. The scaffold protein connector enhancer of kinase suppressor of Ras 2 (CNK2) is predominantly expressed in neural tissues and was recently implicated in X-linked intellectual disability (ID). We have investigated the role of CNK2 in neurons in order to contribute to our understanding of how CNK2 alterations might cause developmental defects, and we have elucidated a functional role for CNK2 in the molecular processes that govern morphology of the postsynaptic density (PSD). We have also identified novel CNK2 interaction partners and explored their functional interdependency with CNK2. We focussed on the novel interaction partner TRAF2- and NCK-interacting kinase TNIK, which is also associated with ID. Both CNK2 and TNIK are expressed in neuronal dendrites and concentrated in dendritic spines, and staining with synaptic markers indicates a clear postsynaptic localisation. Importantly, our data highlight that CNK2 plays a role in directing TNIK subcellular localisation, and in neurons, CNK2 participates in ensuring that this multifunctional kinase is present in the correct place at desirable levels. In summary, our data indicate that CNK2 expression is critical for modulating PSD morphology; moreover, our study highlights that CNK2 functions as a scaffold with the potential to direct the localisation of regulatory proteins within the cell. Importantly, we describe a novel link between CNK2 and the regulatory kinase TNIK, and provide evidence supporting the idea that alterations in CNK2 localisation and expression have the potential to influence the behaviour of TNIK and other important regulatory molecules in neurons.

## Introduction

Scaffold proteins are multi-domain proteins that typically lack enzymatic activity. However, they are crucial in regulating signal transduction cascades: through multiple protein-protein interactions, they organise protein complex formation and ensure spatiotemporal organisation of signalling processes and signal propagation. The scaffold protein *connector enhancer of kinase suppressor of Ras* (CNK/CNKSR) was first discovered in *Drosophila*, where it was shown to regulate Ras/MAPK signalling by binding to the Ras effector RAF and thereby play an essential role in eye and wing development^1,2^. Subsequent studies on CNKs in various organisms showed that CNK homologues are present across species, ranging *e.g*. from *C. elegans*^3^ to humans^1^, and that they likewise influence signalling by acting as scaffolds downstream of Ras^1^. CNKs possess multiple protein interaction domains, including *e.g*. a sterile alpha motif (SAM), a conserved region in CNK (CRIC), a PSD-95/DLG-1/ZO-1 (PDZ) domain, and a pleckstrin homology (PH) domain, and the domain architecture is essentially conserved throughout the CNK family proteins and also across species.

Since the original discovery of the D-CNK protein in *Drosophila*, it has become clear that CNK family homologues serve as scaffolds for multiple signal cascades: By interaction with various guanine nucleotide exchange factors (GEFs) and GTPase activating proteins (GAPs), they modulate signalling mediated not only by Ras but also via Rho family small GTPases^4–6^. It has further been shown that CNKs interact with the cytohesin family of ArfGEFs: CNKs facilitate their membrane recruitment, and thereby regulate the insulin signalling pathway^5,6^. Other studies suggest that CNKs may also regulate cell proliferation and migration by acting as scaffolds directly for the PI3K/Akt^7^ and JNK^8^ signalling cascades. Among the known CNK proteins, mammalian CNK2 isoforms are the proteins that exhibit tissue-specific expression in nervous tissue^9^. There are studies on how CNK2 isoforms participate in protein-protein interactions^9–11^, but relatively few studies focus specifically on the scaffold function of CNK2 in neurons and in the brain, despite an increasing number of genetic studies that directly implicate CNK2 mutations in human brain disorders. Specifically, both deletions^12–15^ and point mutations^16–19^ that result in loss of function of CNK2 cause X-linked intellectual disability (XLID) that is typically associated with seizures. As CNK2 has its highest expression levels in the brain^9^ (see also www.proteinatlas.org), we were interested particularly in the role of the protein in the development and function of neurons. There is evidence supporting a role for CNK2 in regulating GTPase activity in neurons^5^, and a recent high-throughput study suggests that CNK2 may be part of the core scaffold machinery that assembles synaptic signalling complexes in the postsynaptic density (PSD) during development^20^. Understanding the molecular details of its function during development of nervous tissue will contribute to our knowledge about how CNK2 alterations can cause neurodevelopmental disorders.

To explore the function of CNK2, we first examined its specific localisation in neurons and confirmed that it is concentrated at postsynaptic sites^21^. We next utilised an shRNA-mediated knockdown approach to explore the effects of loss of CNK2 function at these postsynaptic sites, *i.e*. in dendritic spines of glutamatergic neurons. We also took advantage of several CNK2 variants and comparatively investigated their properties in heterologous cells and in dendritic spines. Together, these data suggest a functional role for CNK2 in regulation and maintenance of protein content and morphology of dendritic spines. We also identified several protein partners that may participate in the execution of CNK2-mediated regulatory functions in neurons. Subsequent studies focussed on the links between CNK2 and the kinases of the MINK1/TNIK family, for which we discovered a specific interaction with CNK2. We further observed that the subcellular localisation of TNIK is regulated by CNK2 in heterologous cells and in neurons. In summary, our data provide evidence supporting the idea that CNK2 functionally interacts with diverse regulatory molecules in the neuronal environment and that its role as a scaffold is important for regulation and/or maintenance of dendritic spine architecture.

## Results

### CNK2 is a membrane-associated protein expressed in neurons and enriched at postsynaptic sites

**Figure 1:**
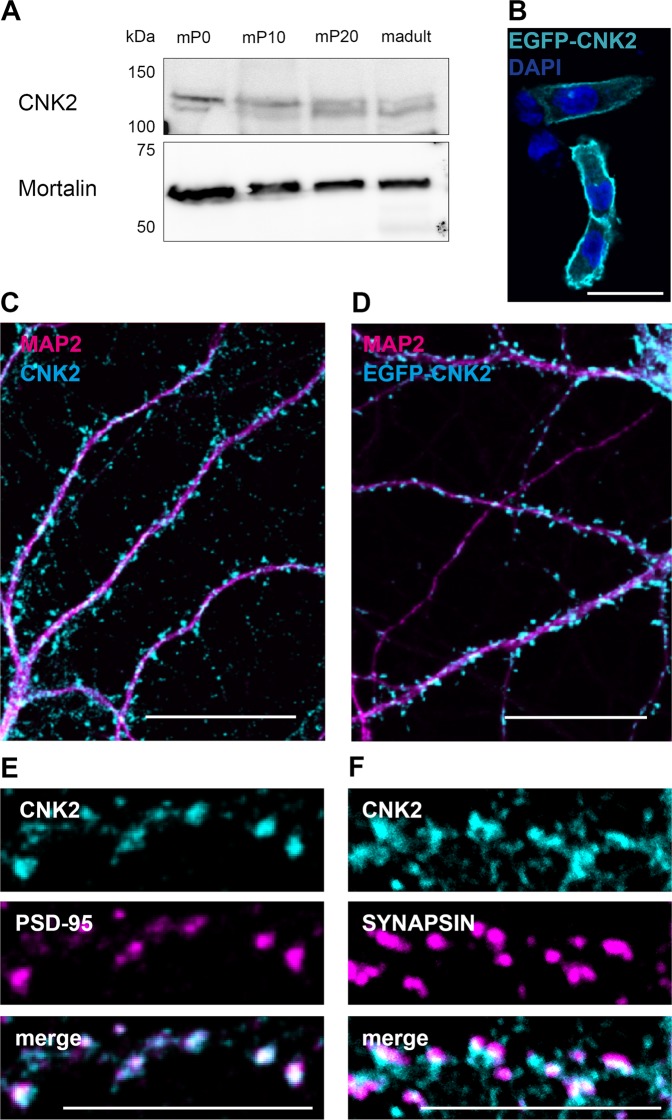
CNK2 is a membrane-associated protein and localised in the post-synapse of neurons. (**A**) CNK2 is expressed from P0 throughout adulthood in the mouse brain. Whole cell lysates generated from mouse brains at P0, P10, P20 and adult stage were analysed by SDS-PAGE and western blot with anti-CNK2 antibody. Mortalin serves as loading control (expected size 60 kDa). (**B**) Membrane localisation of overexpressed EGFP-tagged CNK2 (EGFP-CNK2) in CHL V79 cells (EGFP-CNK2 cyan; DAPI blue). Scale bar: 20 µm. (**C**) Localisation of endogenous CNK2 (cyan) and the dendritic marker MAP2 (magenta) in cultured rat hippocampal neurons (DIV21). Endogenous CNK2 is localised in punctate structures along the dendrite. Scale bar: 20 µm (**D**) Localisation of EGFP-CNK2 (cyan) following lentiviral transduction, and the dendritic marker MAP2 (magenta) in cultured rat hippocampal neurons (DIV21). Scale bar: 20 µm. (**E**) CNK2 (cyan) colocalises with postsynaptic marker PSD-95 (magenta). (**F**) CNK2 (cyan) shows adjacent staining with the presynaptic marker Synapsin (magenta). Scale bar: 10 µm.

As CNK2 is important for early cognitive development in humans, we first assessed its temporal expression in nervous tissue. Based on western blot of mouse brain lysates we could show that the protein is expressed from postnatal day zero (P0) throughout adulthood **(**Fig. 1A**)**. We next explored its subcellular localisation. Following ectopic expression of a tagged variant of CNK2 in CHL V79 cells, we observed a striking membrane-specific localisation **(**Fig. 1B**)**. As the commercially available antibodies did not meet expectations, we produced a custom-made antibody against all known CNK2 isoforms. From immunofluorescence experiments on cultured primary rat hippocampal neurons at DIV21 we could see that endogenous CNK2 is enriched at postsynaptic sites in dendritic spines: it co-localises with PSD-95 and shows adjacent staining with the presynaptic marker Synapsin **(**Fig. 1C,E,F**)**. The postsynaptic localisation of CNK2 was also observed using other methods: following virus-mediated expression of EGFP-tagged CNK2 in hippocampal neurons, we assessed EGFP signal in mature neurons (DIV 21). Again we observed enriched signal intensity in spines **(**Fig. 1D**)**.

### Loss of CNK2 influences the size of the postsynaptic density

**Figure 2:**
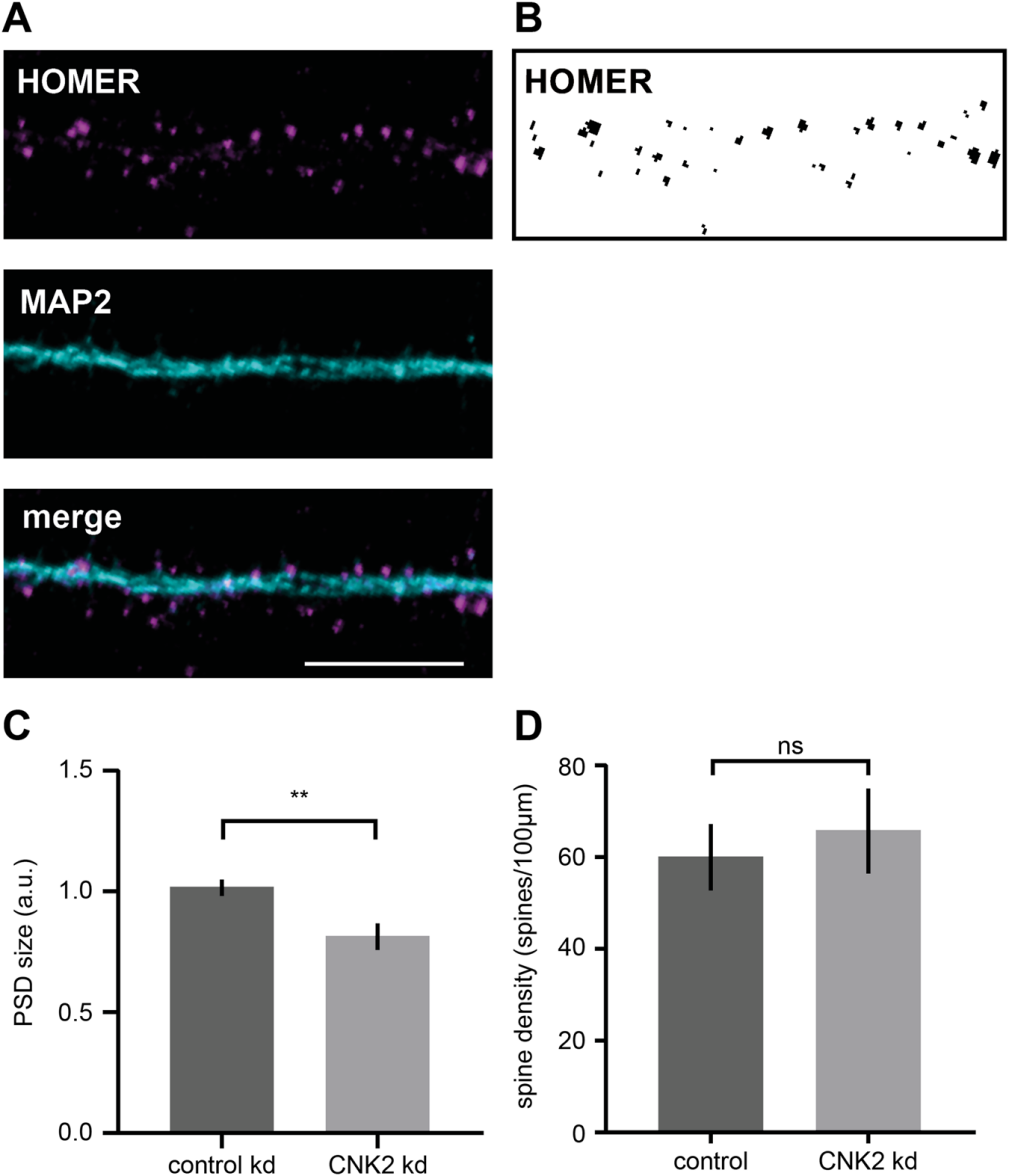
Loss of CNK2 reduces PSD size. (**A**) Cultured rat hippocampal neuron infected with lentivirus transducing shRNA stained for the postsynaptic marker Homer (magenta) and dendritic marker MAP2 (cyan). Scale bar: 10 µm. (**B**) Sample image of endogenous Homer thresholded for analysis of Homer particle size. (**C**) PSD size, represented by the signal of the postsynaptic marker protein Homer (arbitrary units), was analysed using the “Analyze Particles” tool (FIJI/Image). Data were normalised to the median of the control. Graph represents the mean ± SEM (n = 7–10 neurons, N = 3 cultures): control (scrambled shRNA) = 1 ± 0.03, CNK2 knock down = 0.81 ± 0.06; Mann-Whitney test, p < 0.0097. (**D**) Spine density upon lentiviral transduction of scramled control or CNK2 knock down. Homer positive spines were counted. Graph represents the mean of number of spines/100 µm ± SEM (n = 10–13 neurons, N = 4 cultures): control (scrambled shRNA) = 60 ± 7, CNK2 knock down = 66 ± 9; unpaired t-test, p = 0.6501.

Given that complete loss of CNK2 was implicated in brain disorders, together with our observation that CNK2 is expressed in neurons, in particular in dendrites and at postsynaptic sites, we were interested to explore the idea that dendritic spines might be affected by loss of CNK2. We took advantage of lentivirus-mediated gene delivery of an shRNA^5^ to specifically knockdown endogenous CNK2 in primary neurons (Supplement Fig. 1). Eighteen to nineteen days after transduction, neurons were fixed and stained for the postsynaptic marker Homer and the dendritic marker MAP2 **(**Fig. 2A**)**. As a reflection of PSD size, we quantified the endogenous Homer content in mature spines (DIV 21)^22,23^. Blinded analysis of the Homer immunofluorescence signal area using the “Analyze Particles” tool (FIJI/ImageJ)^24^ enabled a quantitative comparative analysis of Homer immunofluorescence signal intensity and area in neurons expressing either CNK2 shRNA or control shRNA **(**Fig. 2B**)**. This analysis revealed a clear reduction of PSD size in CNK2 knockdown neurons (overall reduction of 19%; p < 0.0001) **(**Fig. 2C**)**, and our subsequent comparative analysis of GluA2 intensity in wild-type versus knockdown spines exhibited a similar trend in knockdown neurons (see Supplement Fig. 3). We also comparatively assessed spine density in wild-type and CNK2-knockdown neurons; here we observed no significant differences (see Fig. 2D).

### Expression of a CNK2 variant that does not bind to the membrane affects PSD size

**Figure 3:**
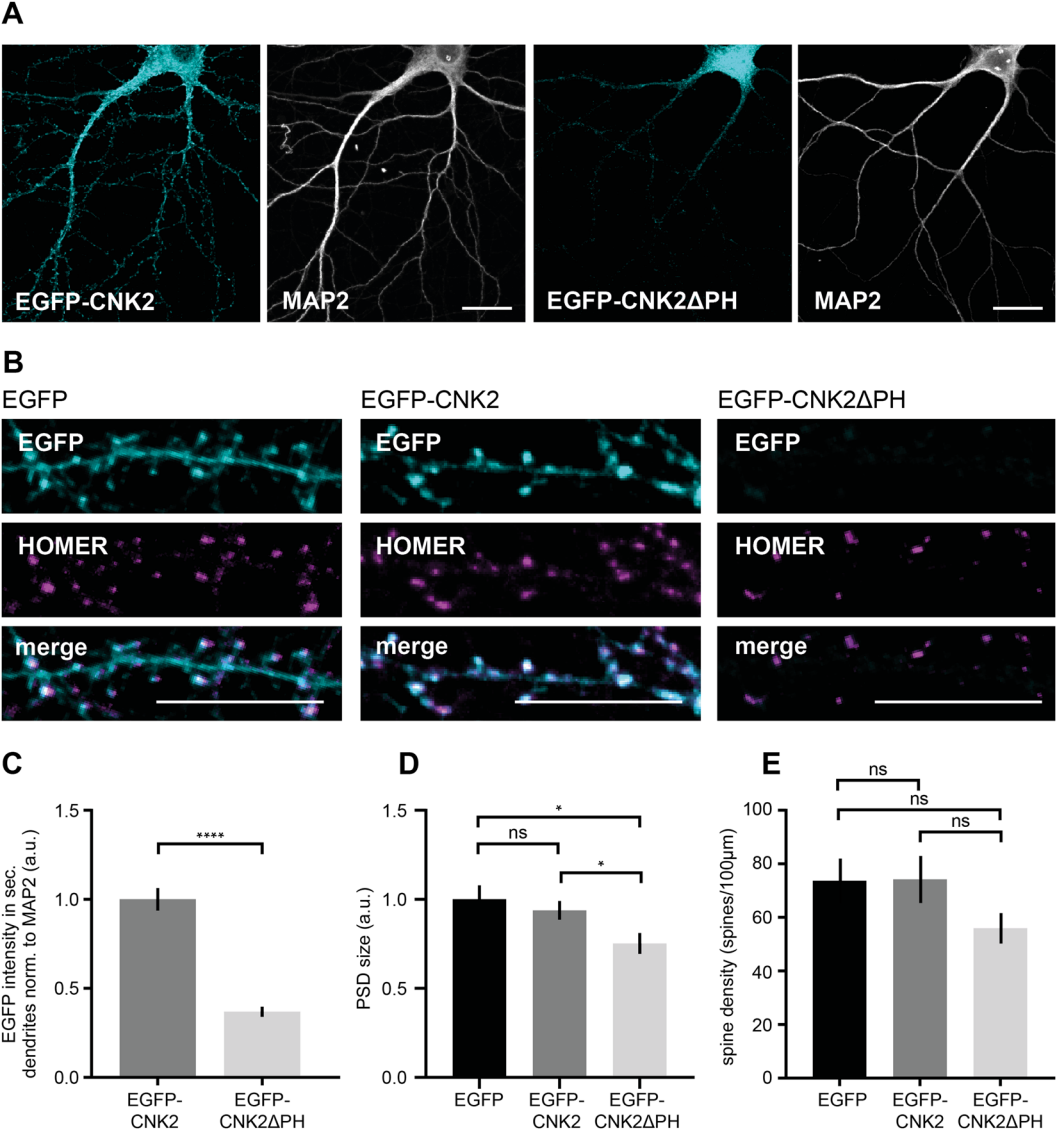
Expression of a CNK2 variant that does not bind to the membrane affects PSD morphology. (**A**) Representative images of primary neurons expressing either EGFP-CNK2 (cyan) or EGFP-CNK2ΔPH (cyan) co-stained for endogenous MAP2 (grey). Scale bar: 20 µm. (**B**) Neurons expressing EGFP, EGFP-CNK2 or EGFP-CNK2ΔPH stained for EGFP (cyan) and Homer (magenta). Scale bar: 10 µm. (**C**) Quantification of amount of EGFP-CNK2 and EGFP-CNK2ΔPH based on EGFP- signal intensity normalised to MAP2 signal intensity in 4–5 regions of interest (ROIs) per neuron along secondary dendrites. Data were normalised to the mean of the control. Graph represents mean ± SEM (arbitrary units, a.u.) of EGFP intensity (n = 11–14 neurons, N = 3 cultures): EGFP-CNK2 = 1 ± 0.06, EGFP-CNK2ΔPH = 0.37 ± 0.02, data were analysed by unpaired t-test, p < 0.0001. (**D**) Postsynaptic density (PSD) size upon expression of EGFP, EGFP-CNK2 or EGFP-CNK2ΔPH. PSD size, represented by the signal of the postsynaptic marker protein Homer (a.u.), was analysed using the “Analyze Particles” tool (FIJI/ImageJ). Data were normalised to the median of the control. Graph represents the mean ± SEM (n = 15–18 neurons, N = 4 cultures); EGFP = 1 ± 0.08, EGFP-CNK2 = 0.94 ± 0.05 and EGFP-CNK2ΔPH = 0.75 ± 0.06; data were analysed by Kruskal-Wallis test, followed by Dunn’s multiple comparisons test: EGFP vs. EGFP-CNK2: p > 0.9999, EGFP vs. EGFP-CNK2ΔPH: p = 0.04; EGFP-CNK2 vs. EGFP-CNK2ΔPH: p = 0.03. (**E**) Spine density upon expression of EGFP, EGFP-CNK2 or EGFP-CNK2ΔPH. Homer positive spines were counted. Graph represents the mean of number of spines/100 µm ± SEM (n = 11–14 neurons, N = 3 cultures): EGFP = 74 ± 8, EGFP-CNK2 = 74 ± 9 and EGFP-CNK2ΔPH = 56 ± 6; data were analysed by Ordinary one-way ANOVA test, followed by Tukey’s multiple comparisons test: EGFP vs. EGFP-CNK2: p = 0.9988, EGFP vs. EGFP-CNK2ΔPH: p = 0.2698; EGFP-CNK2 vs. EGFP-CNK2ΔPH: p = 0.21.

We observed that loss of CNK2 influences PSD size (see Fig. 2). We also validated that wild-type CNK2 is membrane-associated in heterologous cells (Fig. 1B). In order to explore the functional importance of its membrane localisation, we generated an EGFP-tagged CNK2 deletion construct that lacks the C-terminal region including the PH domain. Following ectopic expression of this mutant, we observed a clear loss of membrane association (Supplement Fig. 2), which is in line with biochemical studies indicating that PH domains typically bind to phosphatidylinositol lipids in biological membranes^25^. We expressed this construct in primary hippocampal neurons and compared its expression with EGFP-tagged wild-type CNK2 regarding its influence on PSD size. As done for our CNK2 knockdown neurons, we utilised a quantitative comparative immunofluorescence approach to analyse protein content in dendrites and spines (Fig. 2). We first assessed the general expression of wild-type EGFP-CNK2 and EGFP-CNK2ΔPH in dendrites by comparing EGFP signal intensity relative to the dendritic marker MAP2 (see Fig. 3A, Supplement Fig. 4A), and we observed dramatic differences (for CNK2ΔPH, normalised dendritic signal intensity was approximately one-third that of the wild type; see Fig. 3C**)**. To make sure that this was not an effect of globally reduced expression of the truncated CNK2 variant, we analysed whole cell lysates from neurons overexpressing the CNK2 variants by western blot and observed comparable expression levels for EGFP-CNK2 and EGFP-CNK2ΔPH **(**Supplement Fig. 4B**)**. These observations suggest that this difference does not simply reflect reduced total expression of EGFP-CNK2ΔPH but rather specifically reflects an altered localisation.

Next, we assessed expression of the postsynaptic protein Homer in these neurons in order to assess if expression of modified CNK2 variants likewise influences PSD size (see Fig. 3B**)**. Again, following blinded analysis, we observed a significant reduction of Homer content in the dendritic spines of neurons expressing EGFP-CNK2ΔPH when compared to neurons expressing the wild-type EGFP-CNK2 or EGFP alone **(**Fig. 3D**)**. This result suggests that – as we observe for CNK2 loss of function (Fig. 2) – mis-localisation of CNK2 also interferes with regulation of the size of the PSD. In the same neurons, we did not observe significant differences in the spine density (Fig. 3E).

### CNK2 interacts with TNIK and the two proteins co-localise in dendritic spines

To further investigate the role of CNK2 as a scaffold protein in neurons, we next focussed on novel CNK2 interaction partners. Using a targeted approach based on the idea that specific domains have been shown to be responsible for CNK protein-protein interactions, namely the JNK docking site found in numerous proteins, including CNK family members^26^ and the WW domain found *e.g*. in Vilse/ARHGAP39^5^, we explored the idea that CNK2 might interact with diverse regulatory molecules in our hands. In coimmunoprecipitation experiments in heterologous cells, we showed that CNK2 is indeed capable of interacting for example with the regulatory kinases JNK1 and JNK3 (see Supplement Fig. 5A), presumably via its published JNK docking site^26^. We also observed a previously undescribed interaction between CNK2 and the E3-ubiquitin ligase NEDD4 in similar coimmunoprecipitation assays (see Supplement Fig. 5B). Together, these experiments suggest that CNK2 has the potential to serve as a scaffold for diverse regulatory proteins. In order to identify potentially disease-relevant regulatory molecules that interact specifically with CNK2, we used the full-length CNK2 to perform a Y2H screen of cDNAs from adult mouse brain. The list of interactors identified included proteins with diverse functions, and a significant fraction of positive hits were signalling proteins **(see** Supplement Table 1**)**. Importantly, our list included the Rho-GTPase activating protein Vilse/ARHGAP39, which has previously been shown to interact with CNK2^5^, and thus served as a positive control for our Y2H screen. Two additional proteins identified in our screen, the kinases TNIK and MINK1, were of special interest to us, in part because they have both been implicated in the regulation of neuron structure and glutamate receptor function^27^. In addition, TNIK was recently shown to be associated with intellectual disability in patients^28^, and TNIK knockout mice exhibit cognitive impairment and hyperactivity^29^. Moreover, this protein was recently investigated for its role in the regulation of protein complexes in PSDs^20^.

**Figure 4:**
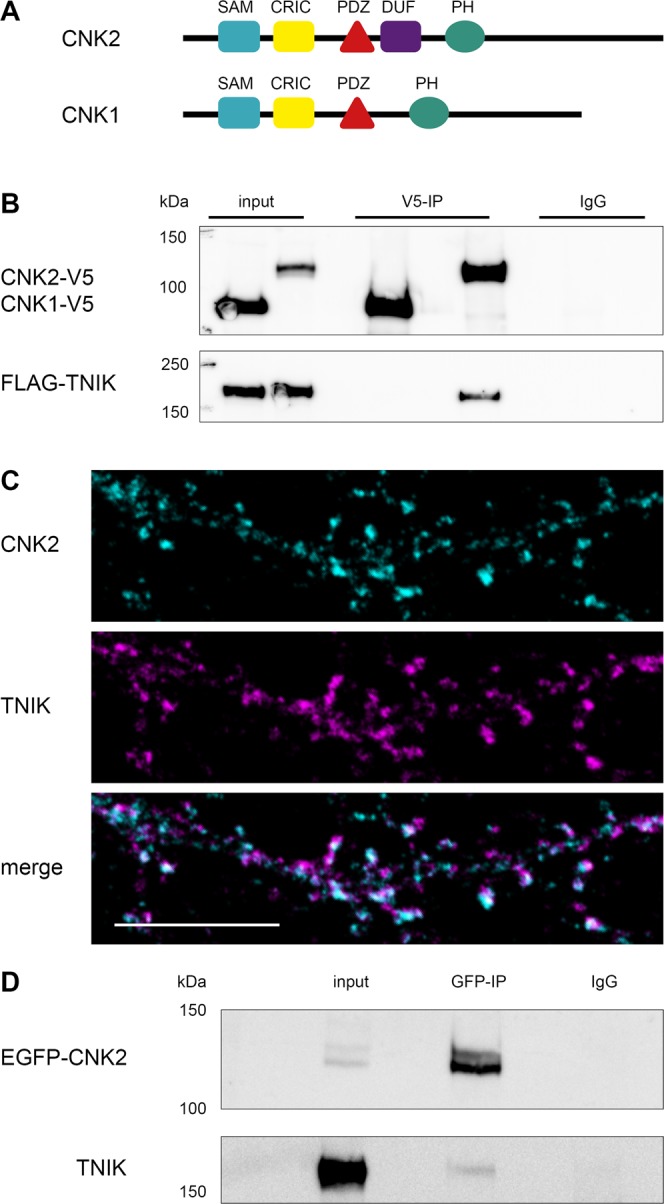
CNK2 interacts with TNIK and the two proteins co-localise in dendritic spines. (**A**) Scheme of CNK1 and CNK2 domain architecture (SAM, sterile alpha motif; CRIC, conserved region in CNK; PDZ, PSD-95/DLG-1/ZO-1; DUF1170, domain of unknown function; PH, pleckstrin homology). (**B**) CNK2 specifically interacts with TNIK. Coimmunoprecipitation experiment with CNK1-V5, CNK2-V5 and FLAG-TNIK expressed in CHL V79 cells. Proteins were immunoprecipitated with either anti-V5 (mouse) antibody or mouse IgGs as a negative control. Proteins were detected by western blot with anti-V5 (CNK2) and anti-FLAG (TNIK) antibodies. (**C**) Representative image of a primary neuron (DIV 21) showing endogenous CNK2 (cyan) and endogenous TNIK (magenta) in dendritic spines. Scale bar: 10 µm (**D**) Coimmunoprecipitation experiment of EGFP-CNK2 with endogenous TNIK expressed in neurons. Protein was immunoprecipitated with either anti-GFP (mouse) or mouse IgGs as a negative control. Proteins were detected by western blot with anti-GFP (CNK2) and anti-TNIK antibodies. Input control (lysate) is shown on the left.

MINK1 and TNIK have a common domain architecture but exhibit minimal sequence homology outside of their conserved domains. We demonstrated that CNK2 interacts with both TNIK and MINK1 in coimmunoprecipitation assays **(**Fig. 4, Supplement Fig. 6**)**. Interestingly, the ubiquitously expressed CNK1 protein, which shares several interaction domains with CNK2 **(**Fig. 4A**)**, did not interact with TNIK or MINK1 in comparable assays **(**Fig. 4B, Supplement Fig. 6A**)**, suggesting that the CNK2-TNIK and CNK2-MINK1 interactions may have CNK2-specific functions that are important in neurons.

Subsequent cell-based studies focussed on the CNK2-TNIK interaction. We first investigated whether CNK2 and TNIK indeed reside in the same subcellular compartments. Using antibodies to the endogenous proteins, we could show by immunofluorescence on cultured primary rat hippocampal neurons (DIV21) that CNK2 and TNIK colocalise in dendritic spines **(**Fig. 4C**)**, providing support for the idea that these two proteins may function together at postsynaptic sites. Using the same antibody to pull down endogenous CNK2, we were not able to observe coprecipitation of TNIK; however, this may reflect antibody-mediated occlusion of the TNIK-CNK2 interaction surfaces. We did confirm that endogenous TNIK can bind to CNK2 in neural tissue: following immunoprecipitation of EGFP-CNK2 expressed in primary rat hippocampal neurons at DIV21, we could detect coprecipitated TNIK by western blot analysis **(**Fig. 4D**)**. Together, these experiments highlight the potential for a functional interaction between CNK2 and TNIK in neurons.

### TNIK localisation is regulated by CNK2

**Figure 5:**
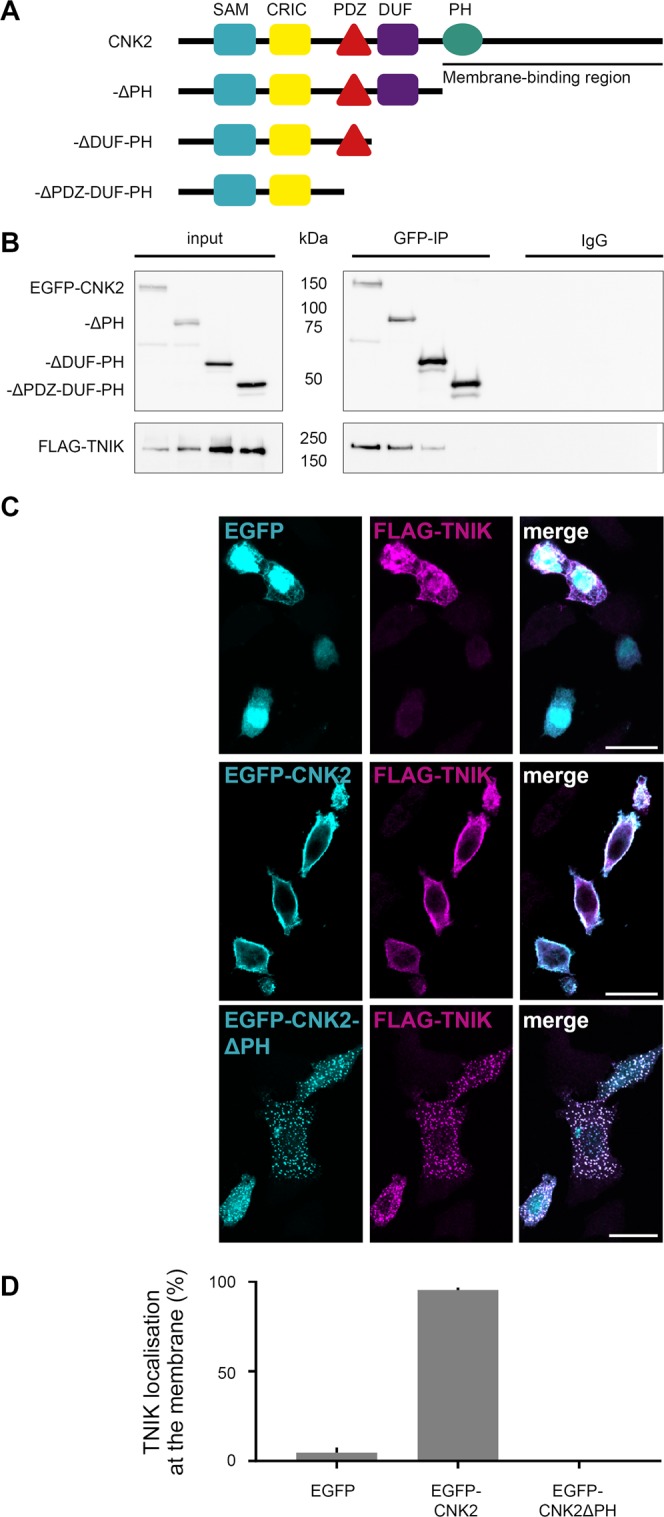
TNIK localisation is regulated by CNK2. (**A**) Scheme of CNK2 truncation variants (for detailed description of domains see Fig. 4A legend). (**B**) Coimmunoprecipitation experiments of EGFP-CNK2 variants overexpressed in CHL V79 cells together with FLAG-TNIK. Proteins were immunoprecipitated with either anti-GFP (mouse) antibody or mouse IgGs as a negative control. Pull-down control and coprecipitated proteins were analysed by western blot with anti-GFP (IP) and anti-FLAG (coIP) antibodies. Input control (lysate) is shown on the left. (**C**) TNIK localisation is influenced by CNK2 in heterologous cells. Representative immunofluorescence experiments in CHL V79 cells overexpressing FLAG-TNIK together with EGFP as control (upper panel), EGFP-CNK2 (middle panel) or EGFP-CNK2ΔPH (lower panel). Left panels show EGFP only or EGFP-tagged CNK2 variants (cyan), middle panels show FLAG-TNIK (magenta), and the right lane shows merged channels. Scale bar: 20 µm. (**D**) For quantification of TNIK localisation in CHL V79 cells reflecting the experiments shown in (**C**), images expressing both proteins, were randomised and classified according to cytosolic or membranous TNIK localisation. Co-expression of CNK2 recruits the main fraction of FLAG-TNIK to the membrane. Upon co-expression of EGFP-tagged CNK2 variants lacking the membrane binding region, FLAG-TNIK shows essentially no membranous localisation. Data used for quantification include data for a total of 73–114 cells per condition, from three independent experiments.

In a subsequent set of experiments, we narrowed down the region within CNK2 relevant for TNIK or MINK1 binding. Using a set of EGFP-tagged CNK2 deletion constructs with C-terminal truncations of various lengths **(**Fig. 5A**)**, we demonstrated via coimmunoprecipitation that loss of the C-terminal region harbouring the PH domain had no effect on TNIK binding. Deletion of both PH and DUF domains, however, did affect binding affinity. Most notably, when the PDZ domain was deleted together with the PH and DUF domains, binding to TNIK and MINK1 was completely abolished **(**Fig. 5B, Supplement Fig. 6B**)**. Together, these results indicate that the region including the PDZ and the DUF domain is critical for CNK2 binding to TNIK and MINK1, and that a CNK2 variant lacking only the PH domain/C-terminus responsible for membrane localisation is still capable of efficient binding to these new interacting proteins.

We next took advantage of this MINK/TNIK-binding and membrane-anchoring-deletion construct (EGFP-CNK2ΔPH) to explore the idea that CNK2 might functionally influence TNIK. We observed earlier that full-length CNK2 exhibits a clear membrane localisation in heterologous cells **(**Fig. 1B**)**. Immunofluorescence of ectopically expressed FLAG-TNIK together with EGFP as a control in CHL V79 cells indicated that TNIK is not enriched at the membrane **(**Fig. 5C, upper panel). However, upon coexpression with full-length CNK2, TNIK was observed at the membrane in most cells **(**Fig. 5C, middle panel), suggesting that CNK2 might play an important role in regulating the subcellular localisation of TNIK. In order to confirm the specificity of this observation we took advantage of the CNK2ΔPH mutant described earlier (see Fig. 5A). Upon cotransfection of EGFP-CNK2ΔPH, TNIK was no longer at the membrane **(**Fig. 5C, lower panel), suggesting that binding to wild-type CNK2 is indeed a mechanism that facilitates TNIK membrane localisation. A comparable experiment with CNK2 and MINK1 indicates that the same is true for this protein-protein interaction (Supplement Fig. 6C), *i.e*. our data suggest that CNK2 is capable of influencing the membrane localisation of both kinases.

We next quantified these results following blinded analysis of TNIK membrane localisation in cells cotransfected with TNIK and either wild-type or mutant CNK2 proteins **(**Fig. 5D**)**. This quantification clearly demonstrates that TNIK localisation is influenced by CNK2 localisation in heterologous cells.

### Mis-localisation of CNK2 also influences TNIK localisation in neurons

**Figure 6:**
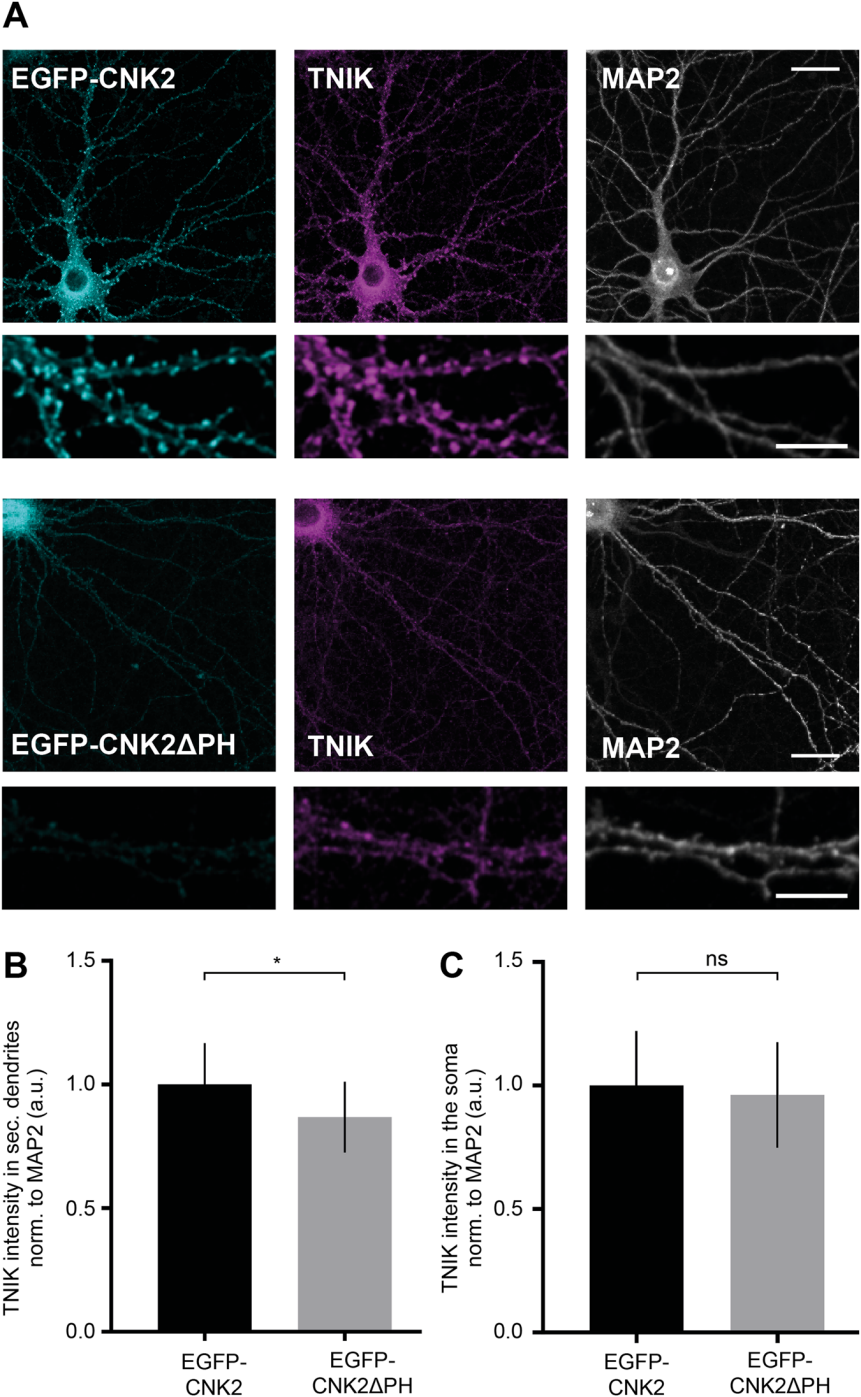
Mis-localisation of CNK2 also influences TNIK localisation in neurons. (**A**) Images of primary neurons and higher magnification image below the corresponding image expressing EGFP-CNK2 (cyan) or EGFP-CNK2ΔPH (cyan) co-stained for endogenous TNIK (magenta) and MAP2 (grey). Scale bar: upper panel: 20 µm, zoom in: 10 µm (**B**) Expression of EGFP-CNK2ΔPH causes a reduction of TNIK in secondary dendrites compared to EGFP or EGFP-CNK2. TNIK intensity was measured in 4–5 ROIs along secondary dendrites and normalised to MAP2 (a.u.). Data were normalised to the mean of EGFP-CNK2. Graph represents mean ± SEM of TNIK intensity (n = 11–14 neurons, N = 3 cultures): EGFP-CNK2 = 1 ± 0.04, EGFP-CNK2ΔPH = 0.87 ± 0.04, data were analysed by unpaired t-test, EGFP-CNK2 vs. EGFP-CNK2ΔPH: p = 0.0478. (**C**) TNIK intensity was measured in the soma of neurons expressing EGFP-CNK2ΔPH or EGFP-CNK2 and normalised to MAP2. Data were normalised to the mean of EGFP-CNK2. Graph represents mean ± SEM (a.u.) of TNIK intensity (n = 11–12 neurons, N = 3 cultures): EGFP-CNK2 = 1 ± 0.06, EGFP-CNK2ΔPH = 0.96 ± 0.06. Data were analysed by unpaired t-test, EGFP-CNK2 vs. EGFP-CNK2ΔPH: p = 0.6782.

To gain further insight into the CNK2-mediated regulation of TNIK in neurons, we examined the precise neuronal localisation of TNIK in neurons expressing either wild-type EGFP-CNK2 or EGFP-CNK2ΔPH. Immunofluorescence of EGFP-tagged proteins in neurons confirmed that – as in heterologous cells – EGFP-CNK2ΔPH exhibited an aberrant localisation in neurons: compared to wild-type CNK2, this truncated version was expressed at reduced levels in dendrites; instead, it accumulated in the soma and in the nucleus, which was not the case for full-length EGFP-CNK2 **(**Supplement Fig. 4A). As we showed earlier **(**Fig. 1**)**, full-length CNK2 is observed primarily in dendrites and in spines. We next assessed TNIK localisation in neurons expressing either full-length or truncated CNK2 variants. Cultured rat hippocampal neurons expressing EGFP-CNK2 or EGFP-CNK2ΔPH were analysed with regard to TNIK content and localisation (Fig. 6A). We normalised endogenous TNIK signal to the signal for the dendritic marker MAP2 and used a quantitative immunofluorescence approach that involved a blinded selection and analysis of the images. On average, the intensity of endogenous TNIK in secondary dendrites was reduced by 13% (p = 0.0478) in neurons expressing EGFP-CNK2ΔPH compared to neurons expressing EGFP-CNK2 **(**Fig. 6B**)**. To explore the idea that this reduction might simply reflect globally reduced TNIK expression in neurons expressing truncated CNK2, we also analysed TNIK intensity in the soma. Here we did not observe significant differences **(**Fig. 6C**)**, suggesting that the reduction we observe might indeed reflect specific loss of dendritic TNIK expression. This result is in line with our data from heterologous cells (Fig. 5). Together, these data suggest that mis-localisation of CNK2ΔPH, which binds to TNIK, is indeed able to influence normal TNIK trafficking to the membrane and thereby also generally modulate its localisation in a cellular environment. Using a similar strategy, we also explored the idea that loss of CNK2 might influence TNIK localisation. We did not observe a significant difference of TNIK content in dendrites following virus-mediated shRNA knockdown of CNK2 in neurons (data not shown), suggesting that other factors also participate in determining the localisation of TNIK in a neuronal environment, and that CNK2-KD-mediated alterations of PSD size are not mediated exclusively through TNIK. Importantly, this observation is consistent with our observations that - despite the dramatic mis-localisation of the CNK2ΔPH variant and corresponding mis-localisation of overexpressed TNIK in heterologous cells (see Fig. 5C,D) where a role for other synaptic proteins can be excluded - endogenous TNIK localisation is only mildly affected by the presence of CNK2ΔPH in the neuronal environment (see Fig. 6B).

## Discussion

Patients with CNK2 mutations exhibit an array of neurocognitive symptoms, ranging from mild intellectual disability and language delay to more severe and general delayed cognitive and motor development. Seizures are also present in most patients^16,19^. Clearly, loss of functional CNK2 has detrimental consequences for normal development of functional neural networks; however, the precise molecular mechanisms by which CNK2 mutations cause disease have not been elucidated to date. Our results indicate that CNK2 is expressed in neurons and enriched at postsynaptic sites. In this study, we also demonstrate that loss or mislocalisation of CNK2 proteins can influence the protein content and size of the PSD. Further studies revealed a set of novel binding partners for CNK2. Interestingly, the proteins identified were not structural proteins that have been previously shown to participate in regulating PSD size. Instead, our list of interacting proteins consisted predominantly of regulatory proteins, including, for example the Rho-GTPase activating protein Vilse/ARHGAP39, which has previously been investigated for its role in mediating CNK2 function^5^, and the regulatory kinases MINK1 and TNIK. We focussed on the new CNK2-interacting kinase TNIK which, like CNK2, was recently implicated in cognitive disorders^28^. TNIK is a well-characterised signalling molecule that plays a decisive role in the activation of multiple signal cascades^30,31^. More recently, it has been shown that it is concentrated in dendritic spines^32^ and that it plays a role, together with MINK1, in the regulation of AMPAR trafficking^27^. We confirmed that CNK2 and TNIK exhibit coexpression in dendrites and at postsynaptic sites, and taking advantage of TNIK-binding CNK2 variants that exhibit aberrant subcellular localisation, we demonstrated that CNK2 mutants directly modulate neuronal TNIK localisation, and thus provide support for the idea that TNIK and CNK2 may participate in common pathways at synaptic sites.

In support of this idea, it has been proposed by others that TNIK is involved in the regulation of synaptic protein degradation^33^. Also relevant is that both p120-catenin and delta-catenin have recently been identified as direct TNIK substrates^31^. Both of these catenins interact with cadherins at synapses, and multiple studies demonstrate that their regulation is an important factor in modulating dendritic spine architecture^34–37^. Moreover, TNIK is an important regulator of JNK signaling^30,38,39^, which may also have implications for regulating synaptic protein content^40,41^. It is thus plausible that alterations in synaptic TNIK expression levels or activity might contribute indirectly to changes in the expression of multiple other synaptic proteins and thereby regulate dendritic spine architecture.

Despite these functional links, in the context of this study we were unable to confirm that the observed CNK2-mediated effects on PSD size are regulated directly through alterations in TNIK. This likely reflects the fact that TNIK is only one of numerous regulatory proteins interacting with CNK2. We show that TNIK localisation is clearly influenced by CNK2, highlighting the role of CNK2 as a membrane-associated scaffold protein for mediating cellular localisation. It is plausible that CNK2 serves this purpose for a diverse set of signalling complexes. In this context, the fact that CNK2 also interacts with Vilse/ARHGAP39 (see Supplemental Table 1 and Lim *et al*.^5^), which has been shown to influence dendritic architecture^42^ (see also Nowak *et al*.^43^, for review), may be of relevance. As is the case for TNIK, the interaction with Vilse/ARHGAP39 relies on residues of CNK2 that are present in the C-terminal half of the molecule. It is possible, therefore, that expression and localisation of Vilse/ARHGAP39 is likewise affected by CNK2 knockdown or expression of a mis-localised CNK2 truncated variant, and that CNK2-mediated morphological effects involve this protein. Additional molecules that may participate in the observed CNK2-mediated effects at the PSD include *e.g*. the regulatory kinase MINK1, which was also identified in our Y2H screen (see Supplement Table 1) and likewise interacts specifically with CNK2 (see Supplement Fig. 6). Interestingly, MINK1 has been shown to act antagonistic to TNIK at synaptic sites^27^, and may therefore neutralise TNIK-mediated effects in response to CNK2 knockdown or mis-localisation.

We also demonstrate in this study that other regulatory molecules, namely JNK family kinases and also the ubiquitin ligase NEDD4, both of which have been shown to influence function and/or morphology at dendritic spines^40,44^, are capable of interacting with CNK2 (see Supplement Fig. 5), highlighting that CNK2 indeed acts as a putative scaffold for numerous signalling modules, several of which could likewise be capable of influencing PSD morphology. In summary, we identify four regulatory proteins as novel interaction partners of CNK2, and based on the example of TNIK, we propose that CNK2 acts as an important scaffold for modulating signalling modules that are potential participants in the regulation of PSD size.

Future studies aim to explore in greater depth how diverse CNK2-mediated signal cascades participate in the cellular processes that govern synaptogenesis and PSD maintenance, and thereby influence neurotransmission and proper network formation.

## Materials and Methods

### Constructs

Cnk2A (Accession number: NM_177751.3) was amplified from cDNA generated from mouse brain. The pEGFP-C1-Cnk2 construct used in this study (Cnk2B, Accession number: NM_001374835.1) was cloned from the Cnk2A cDNA using the primers Cnk2-SalI-fw (aagtcgacatggctctgataatggaaccgg) and Cnk2-BamHI-rv (ttggatccttagcttttctctccaac). For the truncated variants, the forward primer Cnk2-SalI-fw was used together with Cnk2ΔPH-rv (ttggatccttagccaagatcctttca) for pEGFP-C1-CNK2ΔPH, Cnk2ΔDUF-PH-rv (ttggatccttaaagcatgctctgagg) for pEGFP-C1-CNK2ΔDUF-PH and Cnk2ΔPDZ-DUF-PH-rv (ttggatccttattccaggtgagcaga) for pEGFP-C1-CNK2 ΔPDZ-DUF-PH. PCR products were cloned into pEGFP-C1 (Clontech) using BamHI and SalI. EGFP-CNK2 and truncated variants were cloned into a lentiviral shuttle vector under the control of a human synapsin‐1 promoter using BamHI and SalI restriction sites. Mouse Cnk2 was subcloned into pBudCE4.1 using pBud-Cnk2-Notl-fw (aagcggccgcatggctctgataatggaa) and pBud-Cnk2-KpnI-rv (ttggtaccgcttttctctccaacgtt). Rat Cnk1 (accession number: BC099788) was amplified from cDNA-clone (Clone ID: 7934518, Source Bioscience) and subcloned into pBudCE4.1 using the primers rCnk1NotI-fw (aagcggccgcatggagcccgtggag) and rCnk1-BglII-rv (aaagatctgggaggtcaggaggtt).

Vectors expressing FLAG-TNIK and FLAG-MINK1 were generous gifts from Natasha Hussain, (Hussain *et al*., 2011). FLAG MINK1-C (AA 534-1310) was subcloned using the primers Mink1-C-XbaI-fw (aatctagaatgcagcagaactctccc) and Mink1-rv (gtaaccattataagctgc).

### Coimmunoprecipitation, SDS-PAGE and western blot

CHL V79 cells were maintained in DMEM (Lonza) supplemented with 10% FBS (Sigma), 2 mM L- glutamine and penicillin/streptomycin at 37 °C with 5% CO_2_. Transient transfections were done using Lipofectamine 2000 (Invitrogen) according to the manufacturer’s recommendations. Transfected CHL V79 cells (T75 flask) were harvested 18–20 hours post transfection in PBS using a cell scraper and pelleted by centrifugation at 1200 × *g*. CHL V79 cell pellets were lysed in 1 ml lysis buffer (50 mM Tris pH 7.5, 100 mM NaCl, 1% Triton X) and lysed using a 30 G needle. Lysates (1 ml) were cleared by two centrifugations for 10 min at 20000 × *g* and incubated with the appropriate antibody for 3 hours at 4 °C. Lysates were cleared by centrifugation for 10 min at 20000 × *g*. Supernatants were incubated with 30 µl Protein G-Agarose (Roche) per ml lysate for 1 hour at 4 °C and washed three times with lysis buffer. Immunocomplexes were collected by centrifugation, denatured, and analysed by SDS-PAGE and western blot (semi-dry blotting system, Bio-Rad). PVDF-membranes (Bio-Rad) were blocked (PBS, 0.1% Tween 20, 5% dry milk) and incubated overnight with primary antibody. Membranes were incubated for 1 hour at 4 °C with the respective horseradish peroxidase (HRP)-conjugated secondary antibody. If the primary antibody used was conjugated to HRP no secondary antibody was added. Western Lightning Plus-ECL was used to visualise the signal on the blot and recorded with Image Quant (LAS4000Mini, GE Healthcare). To detect other proteins of interest on the same membrane, the membrane was incubated overnight at 4 °C in blocking buffer containing 0.1% sodium acide with subsequent primary and secondary antibody as described before. For coIP from cultured neurons, infected neurons were washed with warm PBS, subsequently lysed in lysis buffer and further treated as described above.

### Neuron culture

For primary rat hippocampal neuronal cultures, embryonic E18 Wistar rats were used. Following decapitation, hippocampi from embryos were isolated and collected in ice-cold DMEM (Lonza). Neurons were separated using Trypsin/EDTA (Lonza) at 37 °C for 5 min. After stopping the reaction with 10% FBS (Biochrom) in DMEM and subsequent washing in DMEM to remove trypsin, the hippocampal tissue was suspended in neuron culture medium (Neurobasal supplemented with B27 and 0.5 mM glutamine) and further dissociated mechanically. For immunofluorescence, neurons were plated onto glass coverslips (d = 18 mm) coated with a mixture of 0.2 mg/mL poly-D-lysine (Sigma) and 2 ug/mL Laminin (Sigma) in PBS at a density of 1.5 × 10^5^ cells per 12-well. For lysates, plates were coated as described before and cells were plated at a density of 7.5 × 10^5^ cells per 6 well. Cell debris was removed after healthy neurons adhered (50 minutes post-plating), and neurons were maintained at 37 °C with 5% CO_2_ in neuron culture medium.

### Lentiviral infection and Immunofluorescence

Cultured neurons on glass coverslips were infected at DIV3 with lentivirus transducing CNK2 shRNA/scrambled control shRNA and at DIV10 for expression of EGFP, EGFP-CNK2 or EGFP-CNK2ΔPH. At DIV23-24, neurons were fixed in 4% PFA in PBS for 10 min. After washing with PBS the cells were permeabilised in 0.2% Triton-X in PBS for 5 min, washed again with PBS and blocked with 4% bovine serum albumin (BSA) in PBS for 1 h at room temperature. Cells were incubated with primary antibodies in 4% BSA in PBS at 4 °C overnight, washed with PBS and subsequently incubated with secondary antibodies in blocking solution. After washes in PBS, coverslips were dipped in deionized water and mounted with Fluoromount-G (Southern Biotech).

### Imaging

Samples were blinded and randomised before analysis. Images were acquired with a Leica laser-scanning confocal microscope (Leica TCS-SP5 II) using the 63x immersion oil objective. Total z-stack range of 2 µm was set with a 0.4 µm inter-stack interval and used in a maximal z-stack projection for further analysis.

### Analysis

Analysis was done blinded and randomised using FIJI/ImageJ software (Version 1.52 g)^24^. For determination of distribution of EGFP-CNK2 and EGFP-CNK2ΔPH (cyan) and its effect on TNIK (magenta), regions of interest (ROI) were defined along secondary dendrites, 4–6 ROIs per neuron. Fluorescence intensity per ROI was measured for all channels. Intensity for EGFP and TNIK signal was normalised to intensity of MAP2 signal (grey, 405) per ROI. Measured EGFP signal intensity of EGFP-CNK2 and EGFP-CNK2ΔPH was normalised to the mean of EGFP-CNK2 signal. For TNIK distribution, every value was normalised to the mean of TNIK in the control situation (EGFP only). For spine analysis, dendrites were imaged as described before. PSD size, represented by the signal of the postsynaptic marker protein Homer was analysed using the “Analyze Particles” tool of FIJI. For each experiment, the threshold for the Homer staining representing the PSD size was set according to experimenters’ discretion. Every spine measured was normalised to the median Homer area of the control condition (EGFP).

### Statistical analysis

All images analysed in this study were blinded during image acquisition, selection of the regions of interest, and analysis. To choose the correct statistical test, data were tested for normal distribution using the D’Agostino-Pearson normality test. Data following Gaussian distribution were analysed using Ordinary one-way ANOVA test followed by Tukey’s multiple comparison test if there were more than two groups, and t-test when two conditions were compared. For data not following normal distribution, we used the Mann-Whitney test for comparing two groups and Kruskal-Wallis test followed by Dunn’s multiple comparison test for comparing more than two groups. For not normally distributed data, the median per cell was defined and normalised to the median of the control condition, and represented in a bar graph as mean of the medians per condition.

### Crude synaptosome preparation

For the crude synaptosome preparation, cultured neurons were treated with Syn-PER Synaptic Protein Extraction Reagent (Thermo Scientific) according to the manufacturer’s recommendations and analysed by SDS-PAGE and western blot.

### Antibodies

Primary antibodies: anti-CNK2 (guinea pig, Eurogentec, custom-made), anti-CNK2 (rabbit, Sigma/Atlas, HPA 001502), anti-Homer-1 (guinea pig, Synaptic Systems, 160004), anti-MAP2 (guinea pig, Synaptic Systems, 188004), anti-MAP2 (mouse, Millipore, 05–346), anti-Mortalin (mouse, Antibodies Inc.,75–127), anti-PSD-95 (mouse, Antibodies Inc., 75–028), anti-SYNAPSIN-1 (rabbit, Synaptic systems, 106 103), anti-TNIK (mouse, Santa Cruz, sc-136103), anti-FLAG-HRP (mouse, Sigma), anti-GFP (chicken, Abcam, ab13970), anti-GFP (mouse, Roche, 11814460001), anti-GFP (goat, Abcam, ab6673), normal mouse IgG (sc-2025, Santa Cruz), anti-V5 (mouse, Invitrogen, R960–25), anti-V5 (rabbit, Millipore, AB3792). Secondary antibodies: anti-mouse-HRP (Dianova, 115-035-003), anti-rabbit-HRP (Dianova, 111-035-003), anti-goat-HRP (Santa Cruz, sc-2020), anti-guinea pig Alexa Fluor 405 (Abcam, ab175678), anti-mouse Alexa Fluor 405 (Invitrogen, A-31553), anti-guinea pig Alexa Fluor 488 (ThermoFisher, A-11073), anti-chicken Alexa Fluor 488 (Jackson Immuno Research, 703-545-155), anti-rabbit Alexa Fluor 568 (Life Technologies, A-11036), anti-mouse Alexa Fluor 568 (Life Technologies, A-11031).

### Custom-made CNK2 antibody

The custom-made CNK2 antibody used in this study was produced by Eurogentec. It was raised in guinea pig against a KHL-conjugated peptide representing CNK2 amino-acids 727–741 and affinity matrix purified. The peptide sequence selected is present an all known CNK2 isoforms.

### Yeast-Two-Hybrid

The Y2H screen (Hybrigenics ULTImate Y2H) was performed by HYBRIGENICS (Paris, France), using full-length CNKSR2 (mus musculus, Gene ID: 245684, aa 1–1032), cloned into pB27 (N-LexA-bait-C fusion) with an adult mouse brain cDNA library.

### Ethical approval

All animals used in this study were treated according to the German regulations and approved by the ‘Landesamt für Gesundheit und Soziales’ (LaGeSo; Regional Office for Health and Social Affairs) in Berlin on the use of animals for research purposes and sacrificed under the permit T0280/10.

## Supplementary information

Supplementary Information.
