# Supplementary material for: Disease-associated synaptic scaffold protein CNK2 modulates PSD size and influences localisation of the regulatory kinase TNIK
Source: Sci Rep. 2020 Mar 31;10:5709. doi: 10.1038/s41598-020-62207-4 (PMC7109135; doi:10.1038/s41598-020-62207-4)
Supplement: Supplementary file 1 — Supplementary Information. [file 41598_2020_62207_MOESM1_ESM.pdf]

Supplement

Disease-associated synaptic scaffold protein CNK2 modulates PSD size and influences localisation of the regulatory kinase TNIK

Hanna L. Zieger[1§], Stella-Amrei Kunde[1], Nils Rademacher[1,2], Bettina Schmerl[1], and Sarah A. Shoichet[1*]

Author affiliations

[1] Neuroscience Research Center, Charité-Universitätsmedizin Berlin, Charitéplatz 1, 10117 Berlin, Germany

[2]German Center for Neurodegenerative Diseases, 10117 Berlin, Germany.

[§]Interdisciplinary Institute for Neuroscience, CNRS, University of Bordeaux, UMR 5297, F-33000, Bordeaux, France (current affiliation)

*Corresponding author and lead contact: sarah.shoichet@charite.de

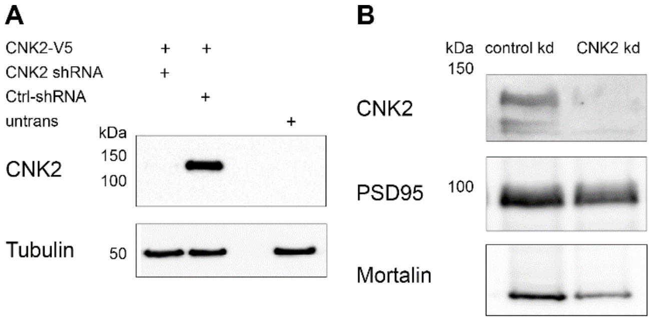

Supplement Figure 1: shRNA-mediated knockdown of CNK2
(A) Western blot of shRNA mediated knockdown of V5 tagged CNK2 expressed in CHL V79 cells. Western blot was probed with anti-V5 antibody. Tubulin serves as loading control. (B) Synaptosome preparation of primary neurons (DIV 23) infected with lentivirus transducing shRNA (DIV 3) to knockdown endogenous CNK2. Control knockdown (left lane) CNK2 knockdown (right lane) tested by western blot with antibody detecting endogenous CNK2 (upper panel). PSD-95 (middle panel) and Mortalin (lower panel) serve as loading control.

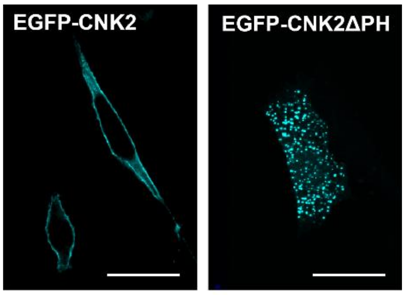

Supplement Figure 2: The CNK2 variant CNK2ΔPH does not bind to the membrane
Image of CHL V79 cells overexpressing EGFP-CNK2 (left) or EGFP- CNK2ΔPH (right). Scale bar: 20µm.

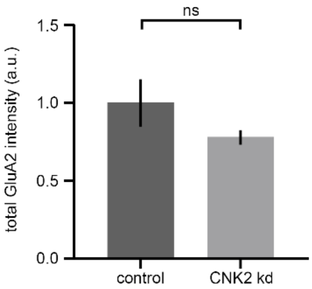

Supplement Figure 3: Analysis of synaptic GluA2 content
Total synaptic GluA2 signal intensity was analysed using the “Analyze Particles” tool (FIJI/ Image). Data were normalised to the mean of the control. Graph represents the mean ± SEM (n= 7-9 neurons, N=2 cultures): control (scrambled shRNA) = 1 ± 0.15, CNK2 knock down = 0.78 ± 0.05; Mann-Whitney test, p = 0.0961

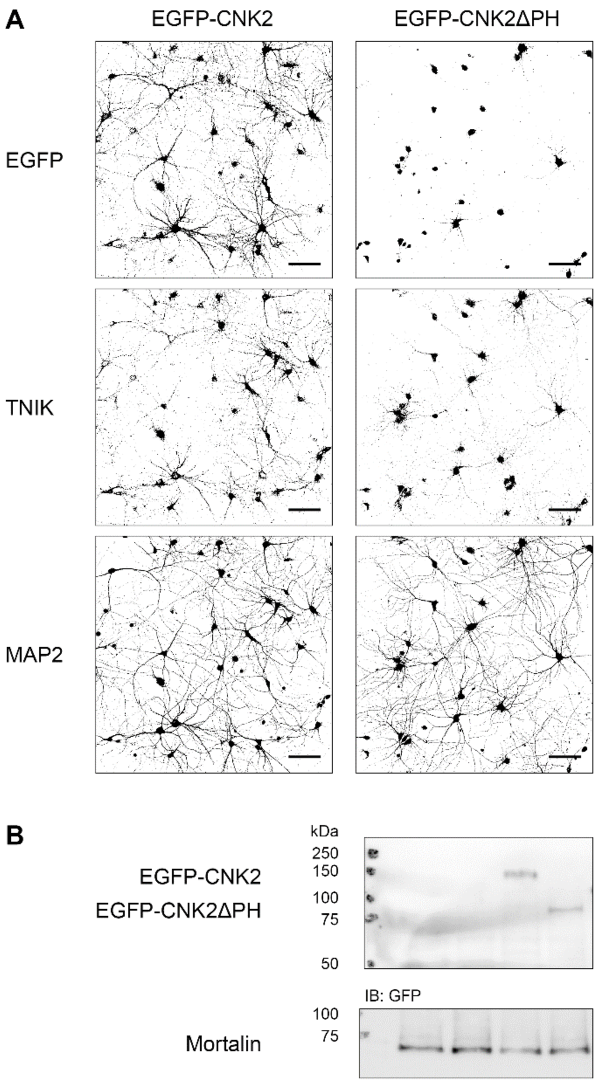

Supplement Figure 4: CNK2ΔPH is mis-localised in neurons

(A) Overview images of neurons (DIV 23) expressing EGFP-CNK2 or EGFP-CNK2ΔPH following virus-mediated gene delivery. Lower panel depicts MAP2, indicating the general dendritic structure of neurons. Thresholds are set identical for each panel (EGFP/TNIK/MAP2). Scale bar: 100 µm. (B) Western blot of whole cell lysates from cultured hippocampal neurons infected with EGFP-CNK2 or EGFP-CNK2ΔPH; proteins detected by western blot with anti-GFP antibody. Mortalin is used as loading control.

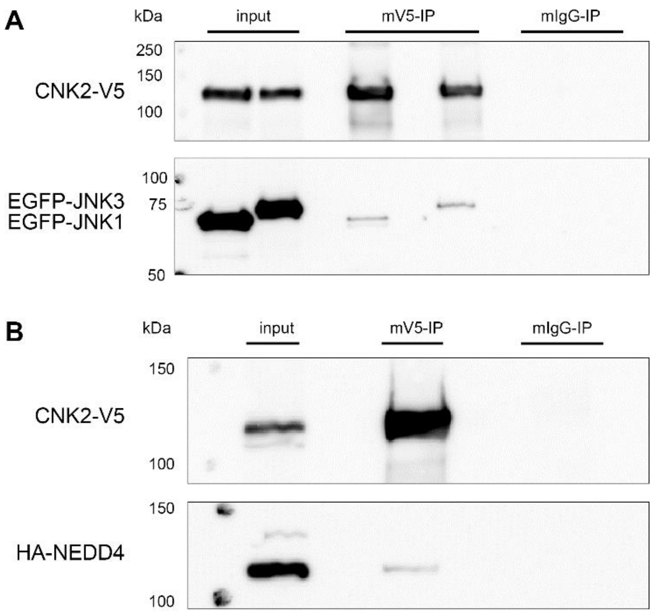

Supplement Figure 5: CNK2 interacts with JNK and NEDD4

(A) Co-immunoprecipitation experiment with CNK2-V5 and EGFP-JNK1 or EGFP-JNK3 expressed in CHL V79 cells. Proteins were immunoprecipitated with either anti-V5 (mouse) antibody or mouse IgGs as a negative control. Proteins were detected by western blot with anti-V5 (CNK2) and anti-GFP (JNK1, JNK3) antibodies. (B) Co-immunoprecipitation experiment with CNK2-V5 and HA-NEDD4 expressed in CHL V79 cells. Proteins were immunoprecipitated with either anti-V5 (mouse) antibody or mouse IgGs as a

negative control. Proteins were detected by western blot with anti-V5 (CNK2) and anti-HA (NEDD4) antibodies.

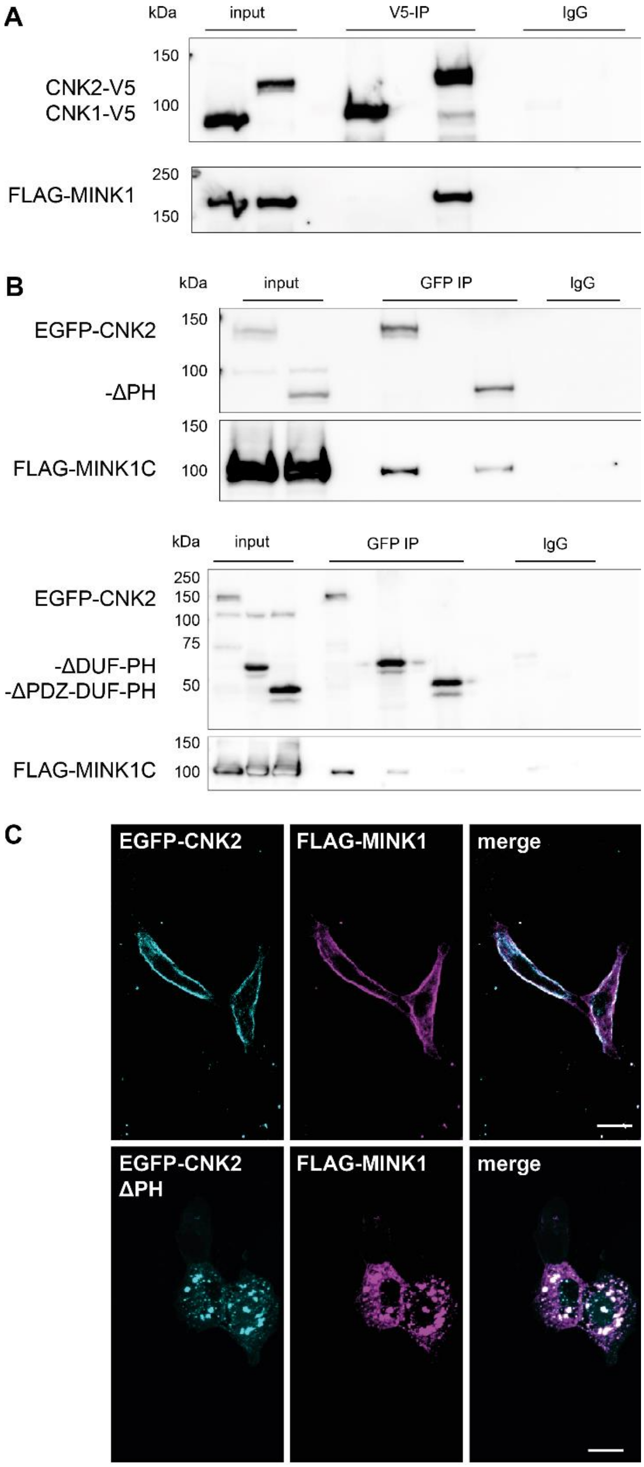

Supplement Figure 6: CNK2 interacts with MINK1 and regulates its localisation

(A) CNK2 specifically interacts with MINK1. Co-immunoprecipitation experiment with CNK1-V5, CNK2-V5 and FLAG-MINK1 overexpressed in CHL V79 cells. Proteins were immunoprecipitated with either anti-V5 (mouse) antibody or normal mouse IgG as a negative control. (B) Co-immunoprecipitation experiments of EGFP-CNK2 variants overexpressed in CHL V79 cells together with FLAG-MINK1C (aa 534-1301). Proteins were immunoprecipitated with either anti-GFP (mouse) antibody or normal mouse IgG as negative control. Proteins were detected by WB. Input control (lysate) is on the left. (C) Immunofluorescence experiment in CHL V79 cells expressing FLAG-MINK1 together with EGFP-CNK2 (upper panel) or EGFP-CNK2ΔPH (lower panel); left lane shows EGFP-tagged CNK2 variants (cyan), middle lane shows FLAG-MINK1 (magenta), and right lane shows merged channels. Scale bar: 10 µm

Gene Name	Gene ID	Interaction domain (AA)	Clones detected
Arhgap39	223666	1-319	2
Cytohesin1	19157	14-100	7
Cytohesin4	72318	6-235	5
Magi3	99470	63-656	1
Mink1	50932	430-533	4
Rlf	109263	989-1496	1
Samd12	320679	41-161	3
Sox5	20678	351-582	1
Tnik	665113	337-598	1

Supplement Table 1: Results from Y2H screen of an adult mouse brain cDNA library

The bait full-length CNKSR2 (*mus musculus*, Gene ID: 245684, aa 1-1032), cloned into pB27 (N-LexA-bait-C fusion), was used as bait to screen with an adult mouse brain cDNA library (by Hybrigenics Services).
